# Duplication of amyloid precursor protein (*APP*), but not prion protein (*PRNP*) gene is a significant cause of early onset dementia in a large UK series

**DOI:** 10.1016/j.neurobiolaging.2010.10.010

**Published:** 2012-02

**Authors:** Daniel McNaughton, William Knight, Rita Guerreiro, Natalie Ryan, Jessica Lowe, Mark Poulter, David J. Nicholl, John Hardy, Tamas Revesz, James Lowe, Martin Rossor, John Collinge, Simon Mead

**Affiliations:** a MRC Prion Unit, Department of Neurodegenerative Disease, UCL Institute of Neurology, Queen Square, London, UK; b Dementia Research Centre, Department of Neurodegenerative Disease, UCL Institute of Neurology, National Hospital for Neurology and Neurosurgery, Queen Square, London, UK; c University Hospital Birmingham, Edgbaston, Birmingham, UK; d Department of Molecular Neuroscience, UCL Institute of Neurology, Queen Square, London, UK; e School of Molecular Medical Sciences, University of Nottingham, Nottingham, UK; f National Prion Clinic, National Hospital for Neurology and Neurosurgery, University College London Hospitals NHS Trust, Queen Square, London, UK; g Laboratory of Neurogenetics, National Institute of Aging, National Institutes of Health, Bethesda, MD, USA; h Centre for Neuroscience and Cell Biology, University of Coimbra, Coimbra, Portugal

**Keywords:** *APP*, Duplication, *PRNP*, Prion, Chromosome 21, Dementia

## Abstract

Amyloid precursor protein gene (*APP*) duplications have been identified in screens of selected probands with early onset familial Alzheimer's disease (FAD). A causal role for copy number variation (CNV) in the prion protein gene (*PRNP*) in prion dementias is not known. We aimed to determine the prevalence of copy number variation in *APP* and *PRNP* in a large referral series, test a screening method for detection of the same, and expand knowledge of clinical phenotype. We used a 3-tiered screening assay for *APP* and *PRNP* duplication (exonic real-time quantitative polymerase chain reaction [exon-qPCR], fluorescent microsatellite quantitative PCR [fm-q-PCR], and Illumina array [Illumina Inc., San Diego, CA, USA]) for analysis of a heterogeneous referral series comprising 1531 probands. Five of 1531 probands screened showed *APP* duplication, a similar prevalence to *APP* missense mutation. Real-time quantitative PCR and fluorescent microsatellite quantitative PCR were similar individually but are theoretically complementary; we used Illumina arrays as our reference assay. Two of 5 probands were from an autosomal dominant early onset Alzheimer's disease (familial Alzheimer's disease) pedigree. One extensive, noncontiguous duplication on chromosome 21 was consistent with an unbalanced translocation not including the Down's syndrome critical region. Seizures were prominent in the other typical *APP* duplications. A range of imaging, neuropsychological, cerebrospinal fluid, and pathological findings are reported that extend the known phenotype. *APP* but not *PRNP* duplication is a significant cause of early onset dementia in the UK. The recognized phenotype may be expanded to include the possibility of early seizures and apparently sporadic disease which, in part, may be due to different mutational mechanisms. The pros and cons of our screening method are discussed.

## 1 Introduction

With the development of experimental therapeutics targeting amyloid-β in Alzheimer's disease (AD) (Salloway et al., 2009) or prion protein in prion disease (Nicoll and Collinge, 2009), diagnosis of dementia in the earliest stages is becoming increasingly important (Burns and Iliffe, 2009). Mutations in any of 4 genes are causative of early onset dementias due to either AD (presenilin 1 [*PSEN1*], presenilin 2 [*PSEN2*], and amyloid precursor protein [*APP*]) or prion disease (prion protein gene, *PRNP*) (Collinge, 2001; Hardy and Orr, 2006). Patients presenting before old age with evidence of a familial disease should be screened for mutations in these genes. However, conventional sequencing will not detect gene deletions or duplications. Over recent years it has been recognized that copy number variations (CNVs), caused by duplication or deletion of parts of chromosomes, are relatively common and occur with a nonrandom distribution across the genome (McCarroll and Altshuler, 2007). The altered regions may include genes that encode for proteins known to be implicated in disease.

The association between AD and Down's syndrome has long been known, providing evidence that a higher gene dosage of *APP* is sufficient to produce an AD phenotype. Recently a number of duplications of small chromosomal regions which include the *APP* locus (*APP*dup) have been reported in 8 French, Dutch, and Japanese families (Cabrejo et al., 2006; Kasuga et al., 2009; Rovelet-Lecruz et al., 2006; Sleegers et al., 2006). The prevalence and phenotypic spectrum of such *APP*dup are yet to be fully defined although the estimated frequency in the selected Rovelet-Lecrux cohort was 8%, about half the contribution of missense *APP* mutations to early onset, autosomal dominant AD (Rovelet-Lecrux et al., 2007). A Finnish family with autosomal dominant early-onset AD and prominent cerebral amyloid angiopathy (CAA) where no causative mutation was identified (Remes et al., 2004) has subsequently been screened, and found to harbor an *APP*dup (Rovelet-Lecrux et al., 2007). While these studies uncovered an important mutational mechanism at *APP*, several questions remain about the frequency of these mutations in larger, less selected *APP* patient cohorts in different countries and the associated phenotypic spectrum. So far as we are aware, *PRNP* CNV in human prion disease has been explored in only 1 series (Collins et al., 2010).

## 2 Methods

### 2.1 Patient cohorts

The entire patient cohort consisted of 1531 samples held at the UCL Department of Neurodegenerative Disease. The AD cohort comprised 873 patient samples referred by the Dementia Research Centre, UCL Institute of Neurology, London. Average age was 57 (standard deviation was 12 years). Three hundred eighty-one of 873 patients had clinically diagnosed AD and sufficient evidence of a genetic cause for their illness to justify diagnostic genetic testing. These 381 samples were screened for mutations in *APP*, *PSEN1, PSEN2*, and *PRNP*. Our local policy is to perform genetic testing on patients with early onset AD if there is evidence from the family history of a genetic cause or in addition, early onset AD patients may be referred for genetic testing if the family history was censored or aspects of the clinical history prompt testing of specific genes. Patients with causal genetic mutations were excluded from the study. Four hundred ninety-two of 873 patients were clinically diagnosed with AD but had not been screened for mutations. These patients had been referred to the Dementia Research Centre, which is a tertiary referral center for cognitive disorders and has a special interest in early onset and genetic diseases, however, aside from this possible source of bias, there were no specified ascertainment/recruitment factors in the AD cohort. The prion disease cohort comprised 658 patients. Two hundred thirty-one of 658 patients diagnosed with probable or definite sporadic Creutzfeldt-Jakob disease (CJD) by the National Prion Clinic, UK; 427 of 658 cases were referred to the National Prion Clinic or MRC Prion Unit for *PRNP* gene testing as a result of prion disease being among the differential diagnoses; average age was 55. Diagnoses in this series were highly heterogeneous or not known to the Department. All patients were *PRNP* mutation negative. All patients gave informed consent for genetic analysis. Ethical approval for the study was given by the University College London Hospitals NHS Trust Local Ethics Committee.

### 2.2 Exon-qPCR, fm-qPCR, and Illumina 610 Bead array

In the exonic real-time quantitative polymerase chain reaction [exon-qPCR], *APP* alleles were quantified on an ABI 7000 Sequence Detection System (Applied Biosystems, Foster City, CA, USA) using the 5′ nuclease assay and duplexed minor groove binding (MGB) probes designed to detect *APP* (exon 5) and *GRN* (chromosome 17, exon 1). In the fluorescent microsatellite quantitative PCR [fm-qPCR] *APP* alleles were quantified by the genotyping of 2 microsatellites located within intron 1 of *APP* (196999; Chr21: 26,460,990–26,461,175) and 330 kilobase (kb) centromeric from *APP* (188463; Chr21: 25,841,358–25,841,530). For details of sample preparation, analysis of *PRNP* and Illumina array (Illumina Inc., San Diego, CA, USA) see supplementary methods.

## 3 Results

### 3.1 Laboratory findings

Using exon-qPCR, comparison was made between the amplification of *APP* or *PRNP* and an internal reference marker (APP-GRN and PRNP-APP respectively). Twenty-one samples from the AD cohort and 19 samples from the prion disease cohort were considered as potential CNV based on whether ΔC_t_ value was greater than 2 standard deviations (2 SDs) from the mean (see supplementary Table 1).

Using fm-qPCR two polymorphic microsatellites within intron 1 and 330 kb centromeric to *APP* and two microsatellites 30 kb and 165 kb telomeric to *PRNP* were amplified using a standard PCR protocol. A comparison of peak area was made in all heterozygous individuals for either one or both of the microsatellite pairs. 6.1 percent of individuals were double homozygotes in the AD cohort and 5.0% individuals in the *PRNP* cohort (see supplementary Table 1); these could not be assessed by this method. Forty-two samples from the AD cohort and 11 samples from the *PRNP* cohort were considered as potential CNVs. Three allelic peaks appeared on the electropherogram of Proband 3 (microsatellite 196,999, see supplementary data).

**Fig. 1:**
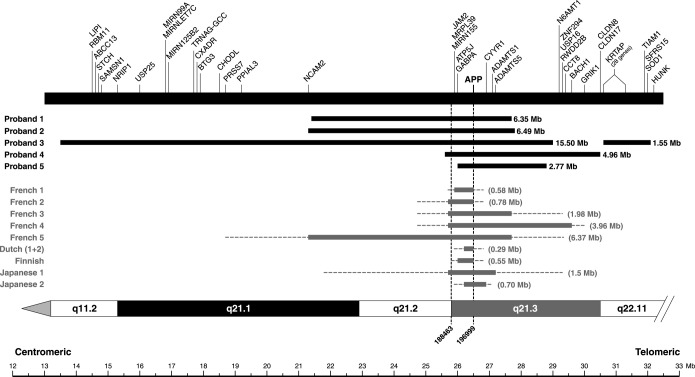
Chr21 diagram showing *APP*dup regions with respect to genes. *APP* duplications in 5 probands. Single nucleotide polymorphism (SNP) start and end positions for these duplications are included in the supplementary data. Black horizontal bars indicate the extent of heterozygous duplications. Minimal sizes (in parenthesis) of previously reported duplications are indicated by gray horizontal bars and the intervals of the duplication boundaries by dotted lines. Gene content excludes pseudogenes and open reading frames. The transcription start site of genes are indicated by vertical bars. Proband 1 has a partial duplication of *NCAM2* and proband 2 has a full duplication of this gene.

No samples from the *PRNP* cohort were positive for both exon-qPCR or fm-qPCR and none were carried forward for verification by Illumina array (Fig. 1 and supplementary Fig. 1). Five samples from the *APP* cohort were positive by both exon-qPCR and fm-qPCR. All these samples were confirmed as heterozygote duplicates including *APP* by the Illumina array. Sixty-three samples were positive by one test. These data were ranked by each individual samples' deviation from the mean, the likelihood of a false positive result for each assay (see supplementary methods), and censored data such as homozygosity of the exon-qPCR microsatellites. With respect to false positive results it is important to consider that analysis of fm-qPCR may be improved by considering the heterozygous microsatellite allele sizes, see supplementary Fig. 2 and legend. From these ranked data we selected the 5/63 thought most likely to be due to duplication involving *APP*. Zero of 5 samples positive on a single assay were confirmed to have *APP* duplication. Importantly, we cannot be certain about the sensitivity of our screening technique as only 5/63 samples positive on a single assay were tested using the Illumina array.

The Illumina 610 Bead array was used for the verification of duplication in samples that were identified as potential CNVs from exon-qPCR and fm-qPCR. These data showed duplicated regions of Chr21q which included the *APP* gene locus in all 5 probands identified as CNVs by both exon- and fm-qPCR. There was considerable heterogeneity in duplication size (2.77, 6.35, 4.96, 6.49 megabases [Mb], see Fig. 1 and supplementary Fig. 1) showing that these patients represented separate duplication events. Although Proband 3 had been identified by fm-qPCR as having three microsatellite alleles, the array revealed this patient to have an extended discontinuous duplication of Chr21q. Coverage of Chr21p with single nucleotide polymorphisms was poor. The large duplicated region is interrupted by a region of unduplicated single nucleotide polymorphisms. In conjunction with the microsatellite haplotyping, these data are consistent with an unbalanced translocation of chromosome 21.

Of the five positive individuals of the AD cohort, 4 (1%), (Probands 1, 2, 3 and 5) originated from the referral series of 381 patients for a diagnostic test of AD gene mutation and screened for *APP*, *PSEN1*, *PSEN2* and 1 (0.2%), (Proband 4) originated from 492 patients comprising a collection of patients thought to have AD but with insufficient clinical evidence to justify screening of causal genes.

### 3.2 Clinical and investigation findings

**Fig. 2:**
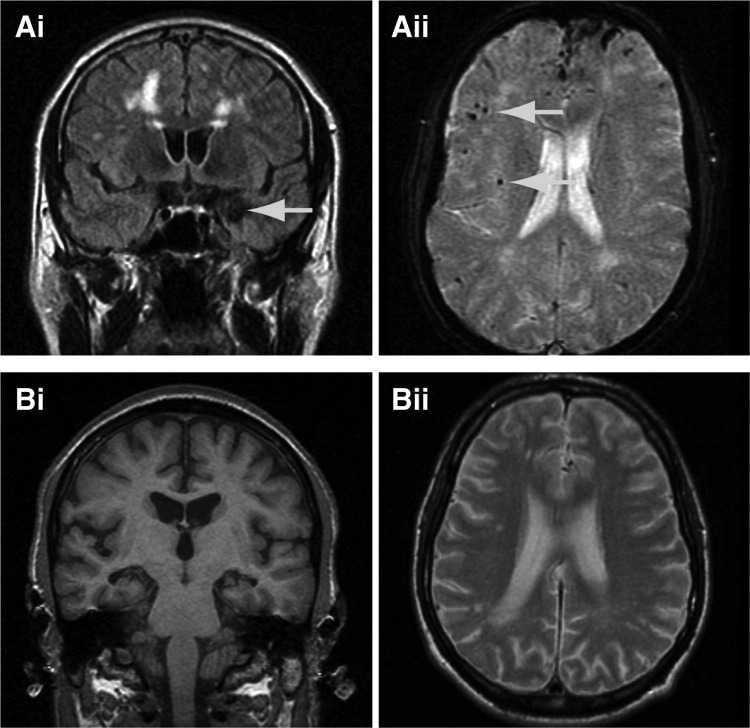
Magnetic resonance imaging (MRI) scans from *APP*dup patients. (A) Proband 1 showing extensive white matter abnormalities, microbleeds, (Aii) and an amygdala hemorrhage (Ai, arrow), and (B) proband 2 (aged 47) showing white matter abnormalities on T2 weighted scans (Bii) and hippocampal atrophy (Bi).

**Fig. 3:**
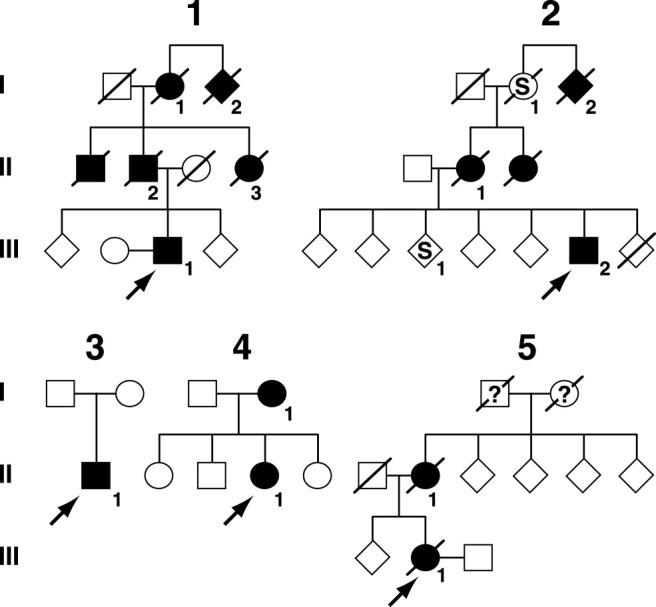
Pedigrees from 5 families. Filled symbols denote affected or probably affected individuals, “S” denotes patient with seizures but not clearly a progressive neurodegenerative disease; see Table 1

**Fig. 4:**
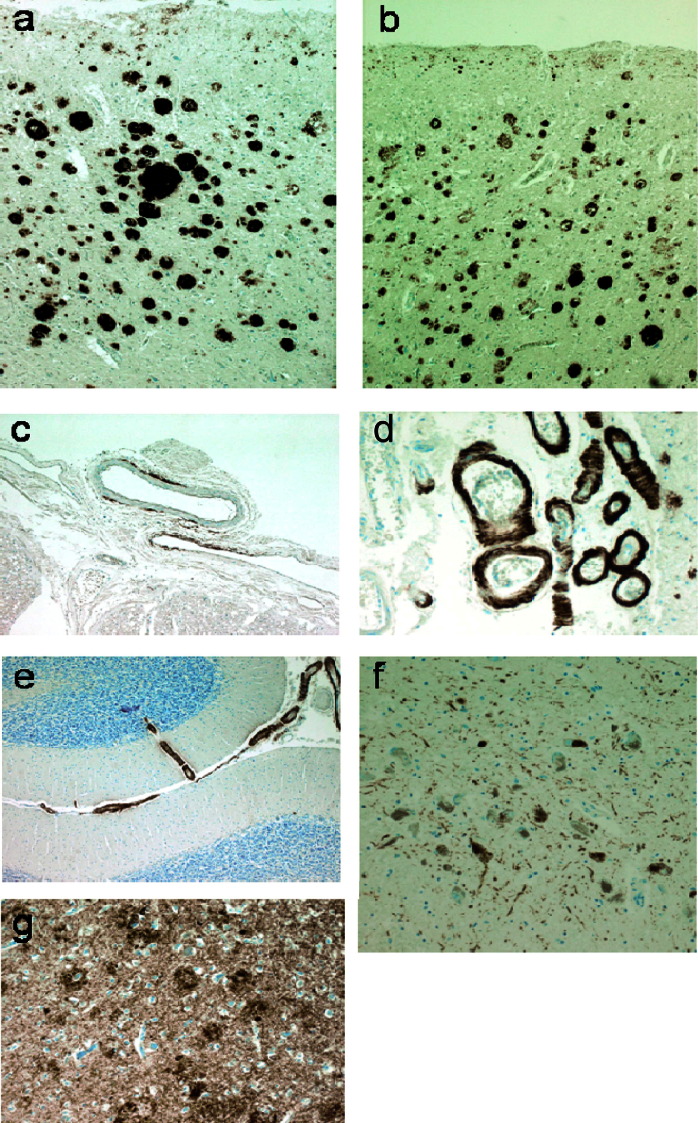
Pathology of proband 4. Amyloid pathology is shown by immunohistochemistry for amyoid beta protein in frontal cortex (a) and temporal cortex (b). Several different patterns of cortical amyloid deposition were seen including diffuse subpial, diffuse, and neuritic plaques and diffuse “clouds” in the entorhinal region. Severe and widespread cerebral amyloid angiopathy was present involving vessels in the spinal (c), cerebral (d), and cerebellar (e) meninges. Rare cerebellar amyloid plaques were noted. Tau pathology was widespread in the temporal cortex (g) mainly in the form of nonplaque neuritic deposition but also including plaque neurites and tangles. Plaque, tangle, and nonplaque neuritic tau deposition were found to affect the cerebral cortex in a wide distribution extending beyond the superior temporal gyrus and heavily involving the occipital cortex amounting to Braak stage VI. Tau staining was prominent in the substantia nigra (f) as both tangle and neuritic deposition. An unusual feature was strong focal glial tau deposition in the white matter in coils and fine neurites (not illustrated). Alpha synuclein staining showed no pathological deposition either in the cortex or subcortical regions including the substantia nigra (not illustrated).

See Table 1 and Figs. 2, 3, and 4.

### 3.3 Proband 1 (ApoE E3, E4)

This 54-year-old man had progressive impairment of episodic memory beginning insidiously at 48 years. Verbal memory was impaired at presentation with general intellectual functions preserved. Later he developed apperceptive agnosia, limb apraxia, and dysexecutive features. At 52 years he had a generalized tonic-clonic seizure. There was an autosomal dominant family history of early onset AD (Fig. 3, Table 1).

Magnetic resonance image (MRI) of the brain showed T2 fluid attenuated inversion recovery (FLAIR) white matter hyperintensities consistent with subcortical vascular disease; multiple small, deep cerebral and cerebellar hemorrhages on T2* imaging, and an old left-sided amygdala hemorrhage (Fig. 2). Electroencephalography (EEG) was unremarkable. Cerebrospinal fluid (CSF) analysis was unremarkable for basic constituents; and showed total tau (t-tau) of 555 pg/mL (upper limit 445) and Aβ-42 of 128 pg/mL (lower limit 427), although normal ranges are not yet conclusively established (Wallin et al., 2006). See supplementary Table 2 for longitudinal neuropsychological assessments.

### 3.4 Proband (ApoE E3, E4) and family 2

This man's family has a history of early onset seizures and midlife cognitive impairment. He developed a variety of seizure types from age 11 years with absences and generalized tonic-clonic seizures. From 40 years there was progressive cognitive decline with social withdrawal, apathy, poor memory, prominent myoclonus, and word-finding difficulties.

Mini Mental State Examination (MMSE) score at 50 years was 10/30. At this stage there was paucity of speech, perseveration, poor concentration, limb dyspraxia, and visuoperceptual difficulties. There was a vertical supranuclear gaze palsy, brisk jaw jerk, and brisk limb reflexes. MRI of the brain revealed severe, generalized cerebral volume loss and bilateral hippocampal atrophy with extensive periventricular white matter signal change. EEG showed an excess of slow wave activity with absent alpha rhythm and bifrontal spike activity. CSF was unremarkable for basic constituents. Longitudinal neuropsychological testing (supplementary Table 2) demonstrated a slow, global cognitive decline.

His sister developed generalized and absence seizures at 11 years. From 50 years she has had some impairment of episodic memory, word-finding difficulties, low mood, and emotional lability although these symptoms have fluctuated and are not clearly progressive. MRI of the brain showed bilateral, periventricular high signal in the deep white matter, basal ganglia and pons, with a high signal area in the right frontal lobe consistent with an old infarct. CSF was unremarkable. Early onset seizures affected his mother, maternal aunt, and sister. The maternal grandmother had seizures, and her sister had seizures and cognitive impairment but no further details are known. Samples were not available for testing of segregation. His mother died aged 64 years with a long history of psychiatric/cognitive problems and generalized epilepsy. Postmortem examination of her brain revealed a previous white matter hemorrhage or infarct with abundant hemosiderin. No vascular amyloid deposition was seen. Diffuse amyloid plaques were present in the striatum only. There were no neurofibrillary tangles.

### 3.5 Proband 3 (ApoE E3, E3)

This Greek man presented at 42 years with a 3-year history of mislaying items and forgetting events. Later problems with route-finding, and social withdrawal were noted. Episodic weeping, and rolling of the eyes with ensuing confusion occurred. These episodes were responsive to anticonvulsant therapy. There was no known family history of dementia.

MRI brain scans revealed generalized atrophy and EEG an absence of alpha rhythm with disorganized activity and delta discharges. CSF analysis was unremarkable for basic constituents. Neuropsychometry revealed difficulty with nonverbal reasoning, dysexecutive features, and impaired episodic memory. Comprehension was intact, word-retrieval problems, and speech production errors (in the form of substitutions, deletions, and transpositions) were evident as well as apperceptive agnosia. Reading, writing, calculation, visuoperceptual, and visuospatial skills were impaired, and limb apraxia was present.

### 3.6 Proband (ApoE E3, E4) and family 4

Little clinical information about this proband is available, although it is known that she suffered from a progressive temporoparietal dementia with late extrapyramidal features (although dopamine antagonist use may have contributed). Age at onset is unknown but by 57 years she was in a nursing home with severe dementia, dysphasia, dyspraxia, motor perseveration, utilization behavior, and wandering. Nothing is known about her siblings but her mother is known to have died aged 49 years from pathologically confirmed “cerebral hemorrhage” and “cystic degeneration of the brain” after a presenile dementing illness.

Proband 4 had a postmortem examination (see Fig. 4).

### 3.7 Proband (APoE E3, E3) and family 5

This 55-year-old woman had progressive memory problems since 48 years when she suffered a focal seizure. Subsequent seizures comprised olfactory aura with focal motor features in the right upper limb. Refractory complex partial and atonic seizures also occurred as well as cognitive decline typical of AD. At 53 years her MMSE was 16/30 and Addenbrooke's Cognitive Examination 50/100. Aged 54 years MMSE was 12/30 and a year later, MMSE was 4/30. Neurological examination was normal.

MRI showed atrophy of the left temporal lobe compared with the right. Tc-Hexamethylpropyleneamine Oxime (HMPAO) single-photon emission computed tomography (SPECT) scan showed irregular diminution of perfusion in the left parieto-occipital region.

The patient's mother died aged 66 years of cancer but had progressive cognitive problems for 5 years including word-finding difficulties and neologisms.

## 4 Discussion

We describe the screening of a large and heterogeneous referral series to identify CNV causing early onset dementia. Five probands with *APP*dup were identified in the screen, 4 with evidence of familial dementia (2 retrospectively) and 1 with a sporadic disease. The overall frequency of mutation in our series of suspected AD patients was 0.57% (95% CI, 0.19–1.33). Although we confirm *APP*dup to be rare, the frequency in this series was comparable to that of missense mutations (5 different *APP* missense mutations have been found from screening the same cohort). The previously reported mutation frequencies in highly selected familial AD were 8% (95% CI, 2.6–17.1) in a French series (Rovelet-Lecrux et al., 2006), 2.7% (95% CI, 0.32–9.3) in a Dutch series (Sleegers et al., 2006) and 0% in Swedish, Belgian, and Finnish cohorts of early onset AD (95% CI, 0–2.58) (Blom et al., 2008). Our data are not directly comparable with more selected studies as 2 from the 5 probands were not identified as having a familial disease until after mutations were identified and this aspect of the history was reconsidered. Only 1 from the 5 probands was considered likely to have CAA because of a scan finding of a small intracerebral hemorrhage (ICH).

In 1 proband (3) we detected a complex discontinuous *APP* duplication mutation associated with sporadic early onset AD. This individual had 3 microsatellite alleles at 1 locus and a double allele dose at the second locus tested. The *APP* duplication patchily involved almost the entire Chr21q, but critically did not include the Down's syndrome critical region around 21q22 (Rahmani et al., 1989). The largest continuous region of duplication was 15.5 Mb. These data are consistent with a different mutational mechanism in proband 3 from the other *APP*dup. This is likely to be an unbalanced translocation similar to partial trisomy of chromosome 21 which is seen in some patients with Down's syndrome (Rahmani et al., 1989). In 4 probands, continuous duplications of 1.6–6.5 Mb including *APP* were detected, 1 mutation was slightly larger than those previously reported.

Although the clinical phenotype of *APP*dup may vary within families, it does not appear to be influenced by the size of the duplication itself (Rovelet-Lecruz et al., 2007), a finding supported by our results. Documented ages at onset range from 40 to 59 years which is in keeping with our data (range 39–61) (Cabrejo et al., 2006; Remes et al., 2004). No clinical features of Down's syndrome have yet been observed although dementia and CAA seem universal, with a quarter also suffering ICH. There was also a high incidence of seizures (57%) in the published series of *APP*dup (Rovelet-Lecruz et al., 2006), and in Down's syndrome (Menendez, 2005). Our data corroborate these reports with a high proportion of patients having seizures in the clinical course. One family (2) has an autosomal dominant history of partial seizures, present from adolescence in patients subsequently affected by a cognitive disorder. Whether these early seizures are coincidental, caused by a linked or unlinked genetic abnormality, or a very early manifestation of CAA is unknown. ICH was not frequent in our series, in contrast to others (Rovelet-Lecruz et al., 2006, 2007), but more consistent with Down's syndrome. Rate of progression and imaging findings were consistent with early onset AD. Clinically it would appear that the *APP*dup-related AD falls somewhere between the canonical AD phenotype observed in most *APP* mutations and the frequent, CAA-associated ICH seen in the Dutch *APP* mutation (Hendriks et al., 1992).

The pathology in our *APP*dup cases may be compared with a limited number of previously published cases (Guyant-Marechal et al., 2008; Rovelet-Lecruz et al., 2006), and larger series of patients with typical AD and Down's syndrome. Features atypical for AD included the severe tau pathology seen in the substantia nigra and marked glial tau pathology in the white matter. As expected, CAA was marked with unusually focal patterns in the spinal meninges, but there was little evidence of microhemorrhage or ischemic damage. The lack of distinctive features of AD in our less well studied case with ICH emphasizes the potential for pathological heterogeneity, however, we were unable to reanalyze these tissues in light of the discovery of a mutation, and it remains possible that the original findings would be revised with more detailed retrospective consideration.

The methods used successfully identified 5 patients where duplication had occurred. Five further samples, ranked from 83 positive using only a single test, were all normal using the Illumina array. Each detection method has its own merits and neither can be used exclusively for the detection of CNV. Although not 100% specific, exon-qPCR cannot be confounded by genomic admixture due to accidental contamination. In contrast, the specificity of fm-qPCR may supersede that of exon-qPCR when one considers the effect of allelic stutter. However fm-qPCR is susceptible to false positive results because of sample contamination and also requires heterozygosity of the marker. The latter issue is largely resolved by typing 2 markers rather than 1. In tandem the techniques are complementary and 100% specific for detecting duplications in our large series. As a further 5 samples, ranked from those positive on only 1 assay, were negative when tested by the Illumina array, it is likely that sensitivity is also very high, although we cannot be certain as only a small number of samples were tested with our reference assay. It remains possible that duplications involving *APP* might be missed when only using a small number of probes, and particularly when the duplicated region is small or if the exon-qPCR probe is homozygous. These methods provide an option with caveats for screening a large sample cohort for this mutation, however further work would be necessary before statements could be made about relative cost-effectiveness or clinical diagnostic accuracy compared with other methods.

The absence of *PRNP* duplication in our large series could indicate either that *PRNP* duplication is an extremely rare cause of dementia, or that *PRNP* duplication is not a cause of human prion disease. Duplication would be expected to increase prion protein (PrP) expression by up to 1.5 times; transgenic mice engineered to overexpress *Prnp* (>8 times) do not develop clinically evident spontaneous prion disease in their normal lifespan, despite having very short incubation times when inoculated with prions (Fischer et al., 1996). A third possibility is that these mutations cause a severe developmental phenotype in humans preventing the ascertainment of later onset prion disease.

Our screening approach allowed us to identify patients with clinical features that would not otherwise prompt consideration of *APP*dup. Selection of patients with an autosomal dominant family history, seizures, or evidence of ICH clinically or on MRI, will identify many, but also miss a large proportion of mutation carriers. This has implications for counseling and diagnosis, though not currently for treatment. However, disease-modifying treatments will demand the ability to identify at risk individuals before irreversible neuronal damage has occurred, justifying a wide screening strategy.

## Disclosure statement

D. McNaughton, W. Knight, R. Guerreiro, N. Ryan, J. Lowe, M. Poulter, D. Nicholl, J. Hardy, J. Collinge, and S. Mead report no disclosures. T. Revesz has received research grant support from Orion Pharma and had a consulting agreement with Merck Serono. He is supported by the Alzheimer's Research Trust and the Sarah Matheson Trust for Multiple System Atrophy and Brain Net Europe. J. Lowe is affiliated with an Alzheimer's Research Trust center supported by an ART center grant. M. Rossor is an NIHR Senior Investigator and SMC for Wyeth/Elan Bapineuzumab study.

All patients gave informed consent for genetic analysis. Ethical approval for the study was given by the University College London Hospitals NHS Trust Local Ethics Committee.

## Figures and Tables

**Table 1:** Clinical features of 11 individuals with definite or possible *APP*dup in the London series

Pedigree	Proband	*APP*dup	Age of onset	Age of death	Clinical features	Seizures	ICH
1III.1	1	Yes	48	—	Memory loss, apraxia, dysexecutive	Late onset tonic-clonic	Yes
1II.1		Not tested	—	63	Dementia	No	—
1II.2		Not tested	Late 40s	62	Dementia	No	—
1II.3		Not tested	—	49	Dementia	No	—
2III.2	2	Yes	40	—	Cognitive decline, memory loss, myoclonus	Early onset tonic-clonic (from age 11)	No
2III.1		Not tested	50	—	Fluctuating memory abnormalities and word-finding difficulty, altered mood	Generalized and complex partial seizures	No
3II.1	3	Yes	39	—	Memory loss, social withdrawal, cognitive decline	Probably complex partial seizures	No
4II.1	4	Yes	—	—	—	—	No
4I.1		Not tested	—	49	Dementia	—	Yes
5III.1	5	Yes	48	—	Memory loss	Late onset generalized and complex partial	No
5II.1		Not tested	61	66	Memory loss, word finding difficulty	No	No

A further 6 family members had some evidence of the typical clinical syndrome of *APP*dup (see family histories and pedigrees). Key: ICH, intracranial hemorrhage.

## References

[bib1] Blom E.S., Viswanathan J., Kilander L., Helisalmi S., Soininen H., Lannfelt L., Ingelsson M., Glaser A., Hiltunen M. (2008). Low prevalence of APP duplications in Swedish and Finnish patients with early-onset Alzheimer's disease. Eur. J. Hum. Genet.

[bib2] Burns A., Iliffe S. (2009). Alzheimer's disease. BMJ.

[bib3] Cabrejo L., Guyant-Marechal L., Laquerriere A., Vercelletto M., De la Fourniere F., Thomas-Anterion C., Verny C., Letournel F., Pasquier F., Vital A., Checler F., Frebourg T., Campion D., Hannequin D. (2006). Phenotype associated with APP duplication in five families. Brain.

[bib4] Collinge J. (2001). Prion diseases of humans and animals: their causes and molecular basis. Annu. Rev. Neurosci.

[bib5] Collins S.J., Schuur M., Coun A.B., Lewis V., Klug G.M., McGlade A., van Oosterhout A., Breedveld G., Oostra B.A., Masters C., Duijn C.M. (2010). No evidence for prion protein gene locus multiplication in Creutzfeldt-Jakob Disease. Neurosci. Lett.

[bib6] Fischer M., Rulicke T., Raeber A., Sailer A., Moser M., Oesch B., Brandner S., Aguzzi A., Weissmann C. (1996). Prion protein (PrP) with amino-proximal deletions restoring susceptibility of PrP knockout mice to scrapie. EMBO J..

[bib7] Guyant-Marechal L., Berger E., Laquerriere A., Rovelet-Lecrux A., Viennet G., Frebourg T., Rumbach L., Campion D., Hannequin D. (2008). Intrafamilial Diversity of Phenotype Associated with App Duplication. Neurology.

[bib8] Hardy J., Orr H. (2006). The genetics of neurodegenerative diseases. J. Neurochem.

[bib9] Hendriks L., van Duijn C.M., Cras P., Cruts M., van Hul W., van Harskamp F., Warren A., McInnis M.G., Antonarakis S.E., Martin J.J., Hofman A., van Broeckhoven C. (1992). Presenile dementia and cerebral haemorrhage linked to a mutation at codon 692 of the b-amyloid precursor protein gene. Nat. Genet.

[bib10] Kasuga K., Shimohata T., Nishimura A., Shiga A., Mizuguchi T., Tokunaga J., Ohno T., Miyashita A., Kuwano R., Matsumoto N., Onodera O., Nishizawa M., Ikeuchi T. (2009). Identification of independent APP locus duplication in Japanese patients with early-onset Alzheimer disease. J. Neurol. Neurosurg. Psychiatry.

[bib11] McCarroll S.A., Altshuler D.M. (2007). Copy-number variation and association studies of human disease. Nat. Genet.

[bib12] Menendez M. (2005). Down syndrome, Alzheimer's disease and seizures. Brain Dev..

[bib13] Nicoll A.J., Collinge J. (2009). Preventing prion pathogenicity by targeting the cellular prion protein. Infect. Disord. Drug Targets.

[bib14] Rahmani Z., Blouin J.L., Creaugoldberg N., Watkins P.C., Mattei J.F., Poissonnier M., Prieur M., Chettouh Z., Nicole A., Aurias A., Sinet P.M., Delabar J.M. (1989). Critical Role of the D21S55 Region on Chromosome-21 in the Pathogenesis of Down Syndrome. Proc. Natl. Acad. Sci. U. S. A.

[bib15] Raux G., Guyant-Marechal L., Martin C., Bou J., Penet C., Brice A., Hannequin D., Frebourg T., Campion D. (2005). Molecular diagnosis of autosomal dominant early onset Alzheimer's disease: an update. J. Med. Genet.

[bib16] Remes A.M., Finnila S., Mononen H., Tuominen H., Takalo R., Herva R., Majamaa K. (2004). Hereditary dementia with intracerebral hemorrhages and cerebral amyloid angiopathy. Neurology.

[bib17] Rovelet-Lecrux A., Frebourg T., Tuominen H., Majamaa K., Campion D., Remes A.M. (2007). APP locus duplication in a Finnish family with dementia and intracerebral haemorrhage. J. Neurol. Neurosurg. Psychiatry.

[bib18] Rovelet-Lecrux A., Hannequin D., Raux G., Le Meur N., Laquerriere A., Vital A., Dumanchin C., Feuillette S., Brice A., Vercelletto M., Dubas F., Frebourg T., Campion D. (2006). APP locus duplication causes autosomal dominant early-onset Alzheimer disease with cerebral amyloid angiopathy. Nat. Genet.

[bib19] Salloway S., Sperling R., Gilman S., Fox N.C., Blennow K., Raskind M., Sabbagh M., Honig L.S., Doody R., van Dyck C.H., Mulnard R., Barakos J., Gregg K.M., Liu E., Lieberburg I., Schenk D., Black R., Grundman M. (2009). A phase 2 multiple ascending dose trial of bapineuzumab in mild to moderate Alzheimer disease. Neurology.

[bib20] Sleegers K., Brouwers N., Gijselinck I., Theuns J., Goossens D., Wauters J., Del Favero J., Cruts M., van Duijn C.M., van Broeckhoven C. (2006). APP duplication is sufficient to cause early onset Alzheimer's dementia with cerebral amyloid angiopathy. Brain.

[bib21] Wallin A.K., Blennow K., Andreasen N., Minthon L. (2006). CSF biomarkers for Alzheimer's disease: Levels of beta-amyloid, tau, phosphorylated tau relate to clinical symptoms and survival. Dement. Geriatr. Cogn. Disord.

