# Supplementary material for: Duplication of amyloid precursor protein (APP), but not prion protein (PRNP) gene is a significant cause of early onset dementia in a large UK series
Source: Neurobiol Aging. 2012 Feb;33(2):426.e13–21. doi: 10.1016/j.neurobiolaging.2010.10.010 (PMC3657692; doi:10.1016/j.neurobiolaging.2010.10.010)
Supplement: Supplementary file 1 — Supplementary data [file mmc1.doc]

SUPPLEMENTARY MATERIAL


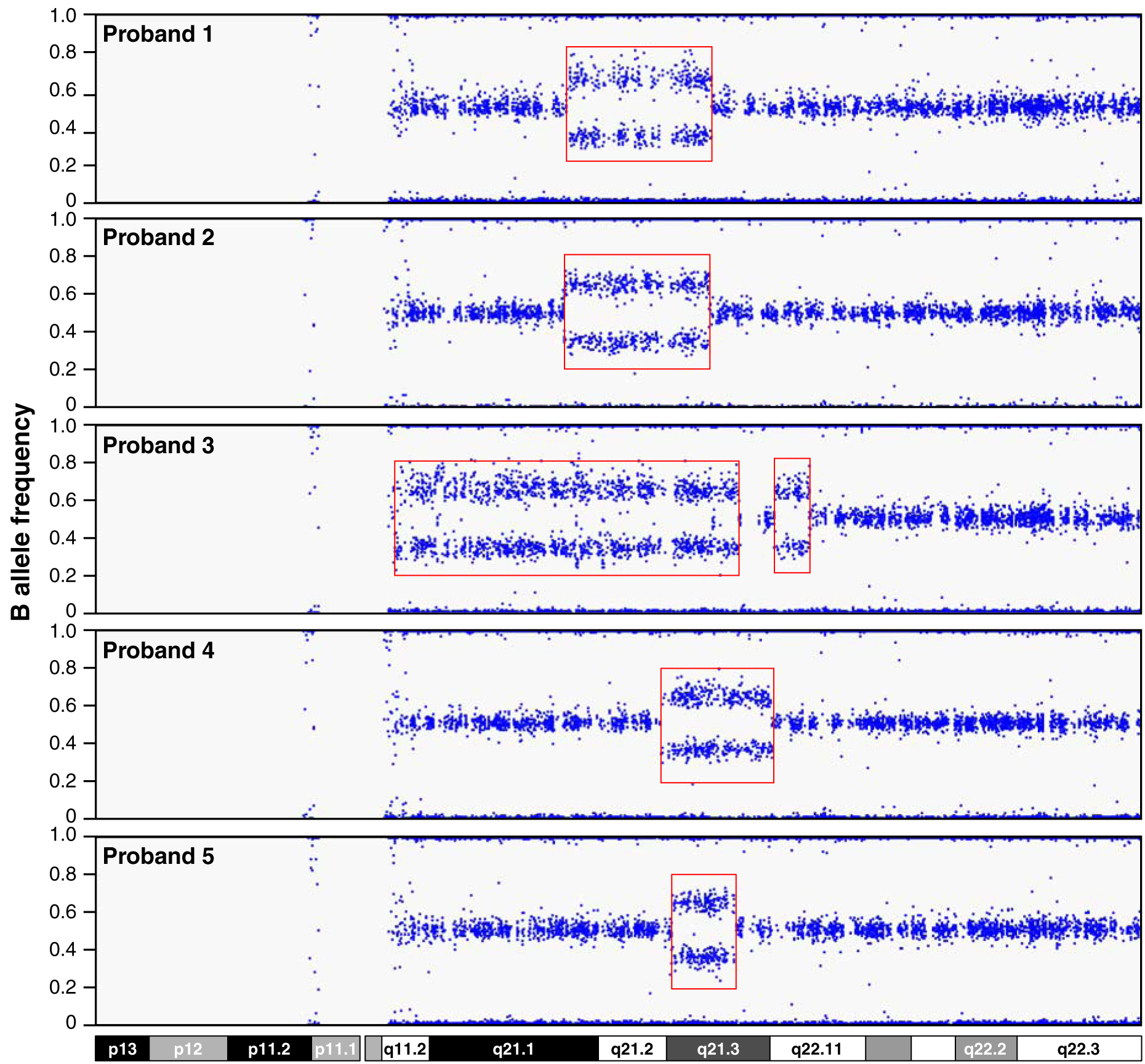


**Supplementary Figure 1. Confirmation of APPdup using Illumina array technology**

Heterozygous duplications in 5 probands. Regions of duplication are highlighted in red boxes. In order to examine individual chromosomes for structural mutations the visualization tool Genome Viewer version 3.2.9 within Beadstudio version 3.1.3.0 (Illumina Inc, San Diego, CA, USA) was used. An ideogram of Chromosome 21 and the B allele frequency is illustrated. 1 pixel=56 KB.


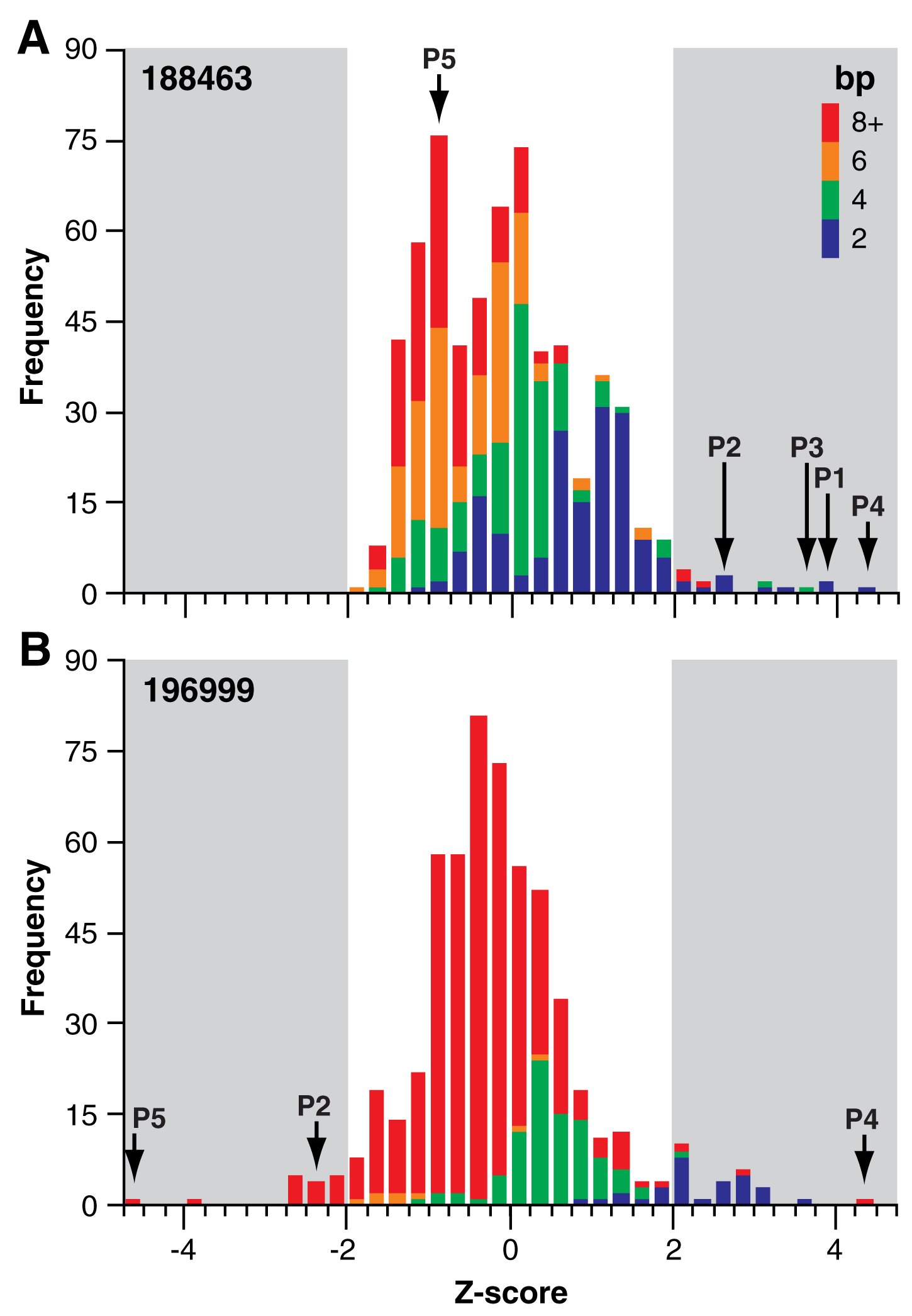


**Supplementary Figure 2. APPdup detection using alteration in the ratio of area under the curve of microsatellite alleles on electropherogram traces.** Histograms are shown to illustrate the distribution of allelic peak area ratios. Variation occurring among the ratios of microsatellite allelic areas appeared to be polymodal. This observation was caused by ‘stutter’ peaks immediately preceeding each microsatellite allele. Microsatellite stutter peaks accentuate the peak of the shorter allele to a degree which is based upon the size differential of the two alleles. Thus, the degree of difference in base pairs between two alleles accounts for part of the variation in allelic ratios. When the two microsatellite alleles are widely separated in length, the ratio is not affected by stutter peaks, whereas when alleles are adjacent the ratio is affected most. This phenomena accounted for most of the false positives using this method alone. Differences in base number between allelic pairs are illustrated by different colour. Shaded areas highlight samples 2SD from the mean. Microsatellite marker 188463 Proband 5 had a normal allelic peak ratio. Array analysis confirmed that this marker lay outside the duplication boundary for this individual. Microsatellite marker 196999 Proband 3 had three microsatellite peaks. And Proband 1 was uninformative due to homozygosity.

|  | **APP CNV Screen** |  |  |  |  |  |
| --- | --- | --- | --- | --- | --- | --- |
|  |  |  |  |  |  |  |
|  | **Total Sample Number** | **Double homozygotes** | **Number Screened** | **Positives** | **True Positive** | **False Positive** |
|  |  |  |  |  |  |  |
| **Exon-qPCR** | 873 | N/A | 873 | 21 | 5 | 16 |
| **fm-qPCR** | 873 | 54 | 819 | 42 | 5 | 37 |
| **Both above tests** | 873 | N/A | 873 | 5 | 5 | 0 |
| **Illumina Array** | 10 | N/A | 10 | 5 | 5 | N/A |
|  |  |  |  |  |  |  |
|  | **PRNP CNV Screen** |  |  |  |  |  |
|  |  |  |  |  |  |  |
|  | **Total Sample Number** | **Double homozygotes** | **Number Screened** | **Positives** | **True Positive** | **False Positive** |
|  |  |  |  |  |  |  |
| **Exon-qPCR** | 658 | N/A | 658 | 19 | 0 | 19 |
| **fm-qPCR** | 658 | 33 | 625 | 11 | 0 | 11 |
| **Both above tests** | 658 | N/A | 632 | 0 | 0 | 0 |
| **Illumina Array** | N/A | N/A | N/A | N/A | N/A | N/A |

Supplementary Table 1. Performance of screening tests for *APP* duplication.

**See methods for an explanation of the different tests used and confirmation using Illumina arrays.**

**Supplementary Table 2 Longitudinal Neuropsychometry**

**Proband 1**

Donepezil was commenced between the 2007 and 2008 assessments.

| DATE(AGE)  **TEST** | **2006(52)** | **2007** |  | **Jan 2008** | **Nov2008** |
| --- | --- | --- | --- | --- | --- |
| **VIQ** | 90 | 87 |  | 88 | Unknown |
| **PIQ** | 76 | 80 |  | 78 | Unknown |
| **RMTwords (%ile)** | CHANCE | <5 |  | <5 | <5 |
| **GNT (%ile)** | 25 | 5-10 |  | 10-25 | 10 |
| **MMSE** | 25 | 23 |  | Unknown | 26 |

Proband 2 Longitudinal Neuropsychometry with corresponding electroencephalographic and imaging profile.

| **YEAR(AGE)**  **TEST** | **1998(44)** | **2000** | **2001** | **2002** | **2003** | **2004(50)** |
| --- | --- | --- | --- | --- | --- | --- |
| **VIQ** | 71 | 78 | 76 | 75 | 62 | 63 |
| **PIQ** | 73 | 67 | 63 | 64 | 62 | 57 |
| **RMTwords** | 29/50 | 34/50 | 27/50 | 16/25* | CHANCE | CHANCE |
| **RMTfaces** | 28/50 | 27/50 | 25/50 | 14/25* | CHANCE | CHANCE |
| **GNT (%ile)** | 10-25 | 10-25 | 10-25 | 10 | 5-10 | 5 |
| **MMSE** |  | 22 |  | 15 | 10 | 10 |
| **MRI** | Frontal white matter lesions.  No regional atrophy | White matter lesions both hemispheres No regional atrophy | White matter lesions both hemispheres Bilateral hippocampal atrophy |  | White matter lesions both hemispheres. Generalized atrophic change, greater anteriorly. Bilateral hippocampal atrophy right smaller than left | Generalized, severe cerebral volume loss. Extensive white matter change more prominent in right hemisphere. Bilateral hippocampal atrophy |
| **EEG** | Frontotemporal slowing | Traces of alpha rhythm, large amounts of theta in both hemispheres |  | | Widespread slowing. No normal rhythms. No posterior alpha rhythm |  |

**VIQ/PIQ** = VERBAL AND PERFORMANCE SUBSCALES OF WAIS-R (WECHSLER 1981)

**RMTw/RMTf** = RECOGNITION MEMORY TEST FOR WORDS AND FACES (*SHORT VERSION) (WARRINGTON EK 1984 & 1996)

**GNT** = GRADED NAMING TEST (MCKENNA & WARRINGTON 1980)

**MMSE** = MINI-MENTAL STATE EXAMINATION (FOLSTEIN 1975)

**Supplementary Methods**

*Sample Preparation*

Genomic DNA (gDNA) was extracted from peripheral whole blood or post mortem brain material using standard techniques. gDNA quality was assessed using agarose gel electrophoresis. Only those gDNA samples of average molecular weight >10Kb were included in the study. Concentration was assessed using a NanoDrop spectrophotometer (ThermoScientific, NanoDrop Products, Wilmington, DE) and samples were diluted to 20ng/μl in 1X tris-EDTA buffer prior to use.

*Real-Time Quantitative PCR (exon-qPCR)*

*APP* alleles were quantified on an ABI 7000 Sequence Detection System (Applied Biosystems, Foster City, CA, USA) using the 5’ nuclease assay and duplexed minor groove binding (MGB) probes designed to detect *APP* (exon 5) and *GRN* (chromosome 17, exon1). Relative quantification of *APP* using *GRN* as the internal reference was determined using the ΔΔCt method. Standard curves (data not shown) showed that all duplexed assays had equal efficiencies, satisfying criteria for the comparative C_t_ method of quantification. Similarly, *PRNP* alleles were quantified using duplexed MGB probes designed to detect *PRNP* (exon 2) and *APP* (exon 5). Primer and probe sequences are available upon request. These tests were designed primarily to demonstrate *APP* or *PRNP* copy number variation (CNV), however, the choice of the internal reference in the screening of individual cohorts allowed for the detection of rare phenocopies by the possible detection of *GRN* CNV in the AD cohort and *APP* CNV in the prion disease cohort respectively. No *GRN* structural variants were identified (Beck *et al* 2008). Twenty nanograms of gDNA were amplified in a 25μl reaction volume containing 1X Expression Mix (Applied Biosystems), and the respective primer (900nM) and probe (200nM) sets for both the gene of interest and the internal reference using the universal protocol. Each sample was quantified in triplicate with each replicate having a different plate reference to minimise the affect of interwell variation. One sample with trisomy 21 was included as a positive control on all plates. All samples with a dosage quotient (expressed as a ΔCT) two standard deviations from the mean were considered as potential CNVs.

*Fluorescent Microsatellite Quantitative PCR (fm-qPCR)*

*APP* alleles were quantified by the genotyping of two microsatellites located within intron 1 of *APP* (196999; Chr21: 26,460,990 – 26,461,175) and 330kb centromeric from *APP* (188463; Chr21: 25,841,358 – 25,841,530). *PRNP* alleles were similarly quantified by genotyping microsatellites located 30Kb (108991;Chr 20: 4,589,668 - 4,589,950) and 165Kb (452; Chr 20: 4,454,279 - 4,454,435) telomeric of *PRNP* (NCBI Map Viewer Build 36.3). Primer details are available on request. Twenty nanograms of gDNA were amplified by denaturation at 95˚C for 1 minute followed by 24 cycles of 95˚C for 30 seconds, 53˚C for 30 seconds and 72˚C for 1 minute. Amplicons were analysed using an ABI 3130xl automated sequencer and GeneMapper software v.4.0 (ABI). In all individuals heterozygous for a given marker the allelic ratio was assessed by relative peak area. Cases were deemed CNV positive by fm-qPCR if allelic ratios were two standard deviations from the mean and if i) both of a microsatellite pair had altered allelic ratio or ii) one of a microsatellite pair had an altered peak ratio with the other genotyped as homozygous or if the other was further from the gene located on the same side of the chromosome.

*Illumina 610 Bead Array*

Samples identified as potential CNV by exon-qPCR and/or fm-qPCR were assayed using the Illumina bead array system according to the manufacturers protocol. All samples were assayed using the Illumina Human610-Quad BeadChips (Illumina Inc, San Diego, CA, USA) as per manufacturer’s instructions, using 200 ng of genomic DNA. These BeadChips assay more than 610.000 tag SNPs and markers, including 60.000 CNV markers, based on the International Human HapMap project release 23 ([www.hapmap.org](http://www.hapmap.org/)). All the samples genotyped had a genotype success rate of more than 99%. In order to examine individual chromosomes for structural mutations we used the visualization tool Genome Viewer version 3.2.9 within Beadstudio version 3.1.3.0 (Illumina Inc, San Diego, CA, USA). Data was analyzed using the Humane Genome Build 35 and two metrics were visualized: B allele frequency and log R ratio. The former is the theta value for an individual SNP corrected for cluster position, which gives an estimate of the proportion of times an individual allele at each polymorphism is called A or B. The log R ratio is the log2 ratio of the observed normalized R value for the SNP divided by the expected normalized R value for the SNPs theta value. Expected R is calculated from the values theta and R, where R is the intensity of dye-labeled molecules that have hybridized to the beads on the array and theta is the ratio of signal at each polymorphism for beads recognizing an A allele to beads recognizing a B allele. The expected R value for any individual at any typed SNP is calculated using a large population of typed individuals. Therefore, the ratio of observed R to expected R in any individual at any SNP gives an indirect measure of the binding efficiency of detected alleles for each polymorphism and thus of genomic copy number. An R above 1 is indicative of an increase in copy number, and values below 1 suggest a deletion. We have shown previously that this is a very reliable method for detecting genomic copy number mutations (Knight *et al* 2008, van de Leemput *et al* 2007, Simon-Sanchez *et al* 2008). We looked at both log R ratio and B allele frequency plots across the whole genome in each sample, with particular interest in the region containing the APP gene, in order to be able to confirm the results previously obtained.

**References**

Beck J, Rohrer JD, Campbell T, Isaacs A, Morrison KE, Goodall EF et al. A distinct clinical, neuropsychological and radiological phenotype is associated with progranulin gene mutations in a large UK series. Brain 2008; 131(Pt 3):706-720.

Knight MA, Hernandez D, Diede SJ, Dauwerse HG, Rafferty I, van de Leemput J et al. A duplication at chromosome 11q12.2-11q12.3 is associated with spinocerebellar ataxia type 20. Hum Mol Gen 2008; 17(24):3847-3853.

Simon-Sanchez J, Scholz S, Matarin MDM, Fung HC, Hernandez D, Gibbs JR et al. Genomewide SNP assay reveals mutations underlying Parkinson disease. Human Mutation 2008; 29(2):315-322.

van de Leemput J, Chandran J, Knight MA, Holtzclaw LA, Scholz S, Cookson MR et al. Deletion at ITPR1 underlies ataxia in mice and spinocerebellar ataxia 15 in humans. Plos Genetics 2007; 3(6):1076-1082.
